# Factors Associated with Spontaneous Passage of Ureteral Stones: A Prospective Clinical Study

**DOI:** 10.5152/tud.2026.26023

**Published:** 2026-09-24

**Authors:** Mehmet Karadayı, Ural Oğuz, Ercan Öğreden, Doğan Sabri Tok, Özay Demiray, Erhan Demirelli

**Affiliations:** 1 Department of Urology, Giresun University School of Medicine, Giresun, Türkiye; 2 Department of Urology, Giresun University Giresun Training and Research Hospital, Giresun, Türkiye

**Keywords:** Ureter, ureteral calculi, spontaneous passage

## Abstract

**Objective:**

The objective of this study was to investigate factors associated with spontaneous passage (SP) of ureteral stones.

**Methods:**

The present prospective study included 113 patients with ureteral stones <1 cm, categorized into SP (n = 60) and non-spontaneous passage (NSP; n = 53) groups. Clinical, laboratory, imaging, and stone-related factors were compared between groups. Multivariable logistic regression was used to assess independent factors associated with spontaneous stone passage.

**Results:**

The median stone size (5.5 mm [interquartile range [IQR] 3.1] vs 7 mm [IQR 3.0]; *P* = .001) and stone burden (20.5 mm^2^ [IQR 29.25] vs 36 mm^2^ [IQR 24.25]; *P* = .01) were significantly lower in the SP group. The mean Hounsfield unit (HU) of the stones was 738 ± 38 HU in the SP group and 884 ± 37 HU in the NSP group (*P* = .007). Fifty-six patients (93.3%) had soft stones in the SP group, and 36 (67.9%) patients had soft stones in the NSP group (*P* = 0.001). A lower C-reactive protein (CRP) level was statistically associated with SP (26.7% vs 50.9%; *P* = .007). Hematuria was also associated with SP (80% vs 62.3%; *P* = .041). In multivariable analysis, stone size remained independently associated with SP (adjusted odds ratio: 0.63 per 1-mm increase; 95% confidence interval: 0.45-0.88; *P* = .007).

**Conclusion:**

Stone composition was not associated with ureteral spontaneous stone passage. Although stone size remained independently associated with SP after adjustment for other relevant variables, stone burden, HU and hardness of the stone, hematuria, and serum CRP level were associated with spontaneous stone passage in univariable analysis. These parameters should be considered when selecting patients for active surveillance of SP.

**Main Points**

- The chemical composition of the ureteral stone does not significantly influence its spontaneous passage (SP).
- Stone size was detected to be independently associated with SP after adjustment for relevant clinical and stone-related factors.
- Contrary to some conventional clinical expectations, the anatomical location and side of the stone, as well as the degree of hydronephrosis, do not have a statistically significant impact on the success of SP.

## Introduction

The lifetime incidence of urolithiasis ranges between 1% and 15%, varying according to age, gender, race, geographical region, climate structure, and occupational groups.^1^ Approximately 20% of the urinary system stones are detected in the ureter.^2^ Stone size strongly influenced the likelihood of SP, with a reported rate of 89% for stones <5 mm, 63.4% for those measuring 5-10 mm, and only 9.1% for stones >10 mm.^3^ Accordingly, current urological guidelines and the existing literature recommend conservative management with expectant follow-up for ureteral stones smaller than 1 cm, allowing SP whenever clinically appropriate.^4^ This approach is also widely adopted in routine urological practice.

In previous studies, several variables have been investigated as potential determinants of SP. In addition to stone size, inflammatory parameters, including C-reactive protein (CRP), procalcitonin, white blood cell (WBC) count, and neutrophil-to-lymphocyte ratio, have also been evaluated for their predictive value.^5-8^

The present study aimed to evaluate stone-related characteristics, including size, area, Hounsfield unit (HU), and degree of hydronephrosis, together with laboratory parameters as potential factors associated with SP of ureteral stones <1 cm. Additionally, for the first time in the literature, we investigated the impact of stone composition on spontaneous stone passage.

## Material and Methods

### Patients

This study was approved by the Clinical Research Ethics Committee of Giresun University (Approval No. KAEK-59, Decision No. 10, Date of Approval: November 7, 2018). Prospective data collection was performed for patients diagnosed with ureteral stones smaller than 1 cm between November 12, 2018, and March 31, 2020. Written informed consent was obtained from all patients who agreed to participate in the study.

During the study period, 642 patients were diagnosed with ureteral stones in the outpatient clinic. Patients were excluded if they were pregnant (n = 3), were younger than 18 years old (n = 16), had a solitary kidney (n = 8), and had grade 4 hydronephrosis (n = 8), chronic kidney disease (n = 12), conditions requiring urgent intervention (n = 49), sepsis (n = 13), or persistent renal colic (n = 54). Additional exclusion criteria included stone size >1 cm (n = 47), renal ectopia (n = 1), ureteral stenosis (n = 1), and ureteral anomaly (n = 1). Patients who declined to participate were also excluded (n = 218).

A total of 211 patients were initially followed up for spontaneous stone passage. Of these, 37 patients underwent ureterorenoscopy (URS) and lithotripsy and were excluded from further analysis. The remaining 174 patients were followed up for 30 days. During follow-up, 99 patients (56.9%) experienced SP. Stone analysis could not be performed in 30 of these patients because the stones could not be retrieved; therefore, these patients were excluded. Seventy-five patients (43.1%) did not experience SP during the follow-up period, and they were referred to surgery of URS. During URS, stone samples could not be obtained for analysis in 22 of these, who were also excluded. Additionally, 9 patients in the SP group were excluded because of missing data. Ultimately, 60 patients with SP constituted the SP group and 53 patients without SP constituted the non-spontaneous passage (NSP) group (Figure 1).

**Figure 1.**
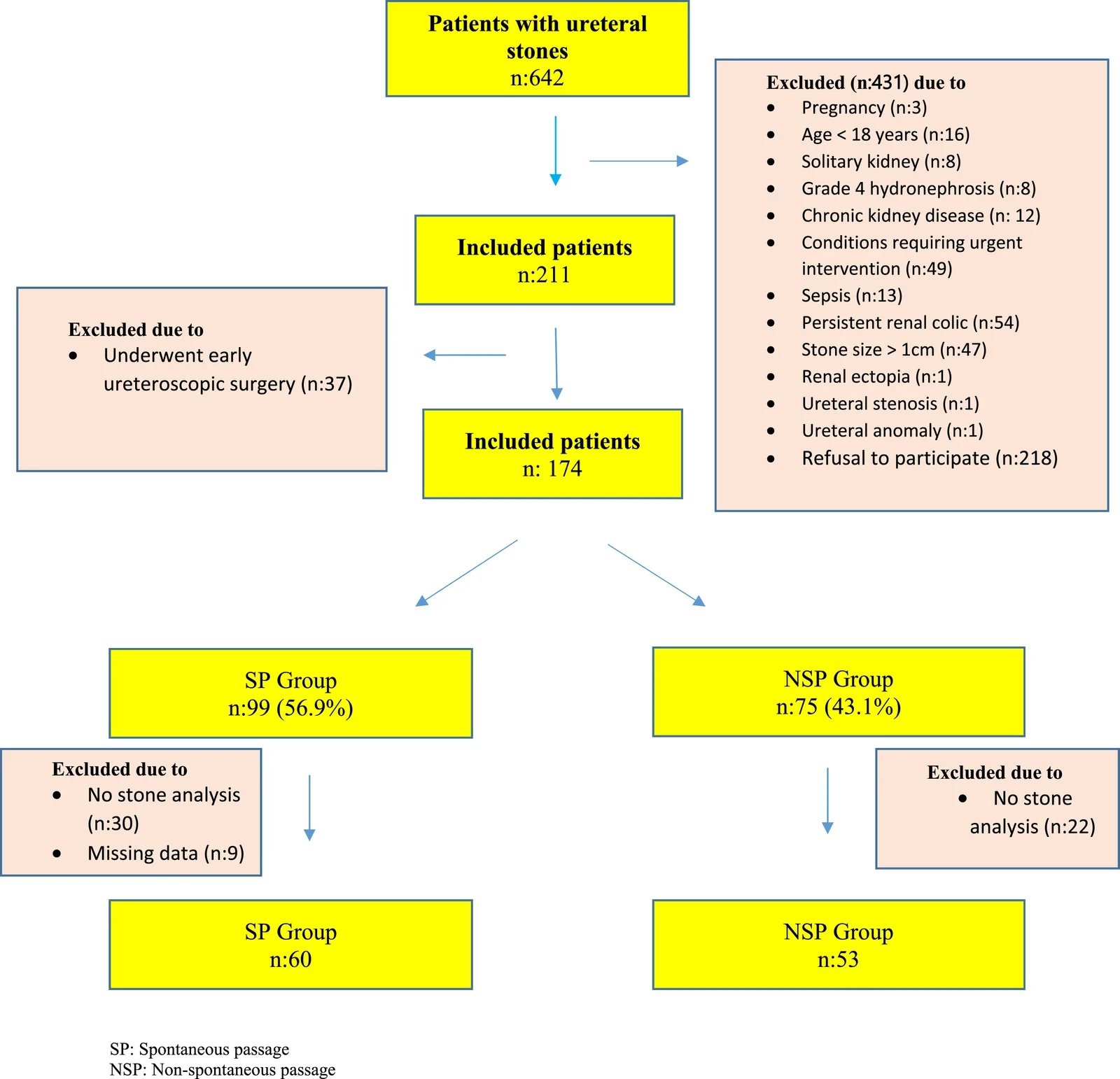
Study flow chart.

### Diagnostic Evaluation and Follow-up

Diagnosis was performed using low-dose non-contrast computed tomography (CT) in 106 (93.8%) patients. We used kidney–ureter–bladder (KUB) radiography and ultrasonography (US) for diagnosis in 7 (6.2%) patients. Routine examinations (full urinalysis, routine biochemistry tests, hemogram, and CRP) of the patients at the time of diagnosis and demographic data of the patients and the stones were recorded prospectively.

Patients with stones smaller than 1 cm were included in the study and were scheduled for follow-up visits at weeks 1, 2, and 4 within a 1-month period. Medical expulsive therapy (MET) was not administered during the follow-up period. Pain was managed with non-steroidal anti-inflammatory drugs (NSAIDs), and patients were advised to maintain adequate oral hydration. Patients with pain refractory to analgesic treatment or an increase in serum creatinine underwent early intervention without waiting for the completion of the 30-day observation period.

Patient age, gender, stone composition and consistency, HU, location, side, size, area, degree of hydronephrosis, presence of leukocyturia, hematuria, blood urea and creatinine, blood leukocyte level, and CRP level were compared between the SP and NSP groups.

### Stone Characteristics and Imaging Parameters

Stone sizes were measured based on the longest axis in KUB, US, and intravenous urography. In CT, the longest axis was taken as the length in the section in which the stone was seen largest, and the product of the 2 longest axes was calculated as the stone area. Hydronephrosis was graded according to the Society of Fetal Urology (SFU) classification, and patients with grade 4 hydronephrosis were excluded from the study.

Ureteral stone location (proximal, middle, and distal) and its side (right, left, and bilateral) were recorded prospectively. A history of spontaneous stone passage and previous URS and shock wave lithotripsy (SWL) treatments were also recorded prospectively.

### Stone Analysis

Stone composition was analyzed using X-ray diffractometry device (D8 Advance, Bruker, Madison, WI, USA). Based on manual fragmentation and crushing characteristics during URS, stones were classified as hard or soft. In addition, stones were categorized by external color as light-colored (yellow or white) or dark-colored (brown or black).

### Laboratory Definitions

The presence of ≥3 erythrocytes in each high-magnification field in the microscopic examination of the complete urinalysis was defined as “hematuria,” and the presence of 5 or more leukocytes was defined as “leukocyturia.”

HU measurement was performed by measuring the smallest 1-mm circular area density, including the geometric and approximate midpoint of the stone, in the CT section in which the stone was seen clearest and widest. This value was recorded as the HU of the stone.

### Statistical Analysis

All statistical analyses were performed using SPSS 21.0 software (SPSS Inc, Chicago, IL, USA).

The normality of continuous variables was assessed using the Kolmogorov–Smirnov test. Data are summarized as mean ± SD for normally distributed variables and as median (interquartile range [IQR]) for non-normally distributed variables. Continuous variable were compared using Student’s *t* test or the Mann–Whitney *U* test, as appropriate, whereas categorical variables were compared using the chi-square test. A *P* value of less than .05 was considered statistically significant.

Factors independently associated with SP were assessed using multivariable logistic regression, with SP as the outcome. SP was defined as the dependent variable. Stone size, HU, elevated CRP (>0.5 mg/dL), hematuria, stone location, and age were included as covariates based on their clinical relevance and the findings of the univariable analyses. Stone area was not included in the same model as stone size because of the high correlation between these variables, to avoid multicollinearity. Effect estimates are reported as adjusted odds ratios (ORs) with 95% confidence intervals (CIs). A *P* value < .05 was considered statistically significant.

This study used ChatGPT to improve language in some parts of the manuscript. However, all content of the study has been finally reviewed and edited by the authors.

## Results

The mean age was 42.6 ± 1.9 years in the SP group and 47.1 ± 2.1 years in the NSP group, with no significant difference between the groups (*P* = .122). Female-to-male ratios were 0.33 and 0.47 in the SP group and the NSP group, respectively (*P* = .266).

The median time to spontaneous stone passage was 9 days in the SP group.

Patients in the SP group had a significantly smaller median stone size than those in the NSP group (5.5 mm [IQR 3.1] vs 7 mm [IQR 3], respectively, *P* = .001). Similarly, the median stone area was significantly lower in the SP group than in the NSP group (20.5 mm^2^ [IQR 29.25] vs 36 mm^2^ [IQR 24.25]; *P* = .01).

Regarding stone location, proximal ureteral stones were detected in 13 (21.7%) patients in the SP group and 17 (32.1%) patients in the NSP group. Middle ureteral stones occurred in 2 (3.3%) and 4 (7.5%) patients in the SP and NSP groups, respectively, while distal stones occurred in 45 (75%) and 32 (60.4%) patients, respectively (*P* = .12).

While 1 (1.7%) patient had bilateral stones, 26 (43.3%) had right-sided stones and 33 (55%) had left-sided stones in the SP group. In the NSP group, right-sided stones were observed in 23 patients (43.4%), left-sided stones were observed in 29 patients (54.7%), and bilateral stones were observed in 1 patient (1.9%) (*P* = .995).

Ureteral stones were diagnosed up on CT examination in 54 patients (90%) in the SP group and 52 (98.1%) patients in the NSP group. The mean density was 738 ± 38 HU in the SP group and 884 ± 37 HU in the NSP group, with a significantly lower value in the SP group.

The presence and degree of the hydronephrosis were similar between the groups (*P* = .462). Previous URS was reported in 16% of the SP group and 7.5% of the NSP group (*P* = .111). A previous history of spontaneous stone passage was reported in 20 patients (33.3%) in the SP group and 16 patients (30.2%) in the NSP group (*P* = .366). Data on previous SWL were not analyzed, as no patient in the NSP group had a history of SWL.

Hematuria was detected in 48 patients (80%) in the SP group and 33 patients (62.3%) in the NSP group (*P* = .041). Hematuria occurred more frequently in the SP group, whereas leukocyturia rates were comparable (16 [26.7%] vs 16 [30.2%] patients; *P* = .393).

Median serum CRP levels did not differ significantly between the SP and NSP groups (0.24 mg/dL [IQR 0.49] vs 0.36 mg/dL [IQR 0.72]; *P* = .172). In contrast, elevated CRP was more common in the NSP group than in the SP group (27 [50.9%] vs 16 [26.7%] patients; *P* = .007).

Median blood leukocyte counts were 9100 (IQR 3.75) in the SP group and 8100 (IQR 4) in the NSP group, with no significant difference between the groups (*P* = .368). Serum calcium, creatinine, and urea levels were also comparable between the 2 groups (*P* > .05).

The rate of soft/medium hardness and light-colored stones was significantly higher in the SP group (*P* = .001). However, stone composition did not differ between the groups. Calcium oxalate stones were detected in 36 patients (60%) in the SP group and 32 patients (60.4%) in the NSP group (*P* = .561) (Table 1).

**Table 1.** Data of the Groups

Variables	Group SP		Group NSP		All		Chi-Square Test	
	n	%	n	%	n	%	Chi-Square	*P*
Gender								
Female	15	25	17	32.1	32	28.3	+	.266
Male	45	75	36	67.9	81	71.7		
All	60	100	53	100	113	100		
Stone Type								
Calcium oxalate	36	60	32	60.4	68	60.17	+	.561
Others	24	40	21	39.6	45	39.82		
All	60	100	53	100	113	100		
Number of Stones								
One stone	52	86.7	50	94.3	102	90.26	+	.146
Two stones	8	13.3	3	5.7	11	9.74		
All	60	100	53	100	113	100		
Previous URS								
Existent	10	16.7	4	7.5	14	12.5	+	.111
Absent	49	81.7	49	92.5	98	87.5		
All	59	98.4	53	100	112	100		
Previous Spontaneous Stone Passage								
Existent	20	30.3	16	30.2	36	32.72	+	.366
Absent	37	61.7	37	69.8	74	67.27		
All	57	92	53	100	110	100		
Hematuria								
Existent	48	80	33	62.3	81	72.32	+	**.041**
Absent	12	20	19	35.8	31	27.67		
All	60	100	52	98.1	112	100		
Pyuria								
Existent	16	26.7	16	30.2	32	28.57	+	.393
Absent	44	73.3	36	67.9	80	71.42		
All	60	100	52	98.1	112	100		

NSP, non-spontaneous passage; SP, spontaneous passage; URS, ureterorenoscopy.

Bold values indicate statistical significance (*P* < .05).

Multivariable logistic regression showed that stone size remained independently associated with spontaneous stone passage (adjusted OR: 0.63 per 1-mm increase; 95% CI: 0.45-0.88; *P* = .007). The odds of SP decreased with increasing stone size after controlling for HU, elevated CRP, hematuria, stone location, and age. Hematuria showed a trend toward an association with SP (adjusted OR: 2.63; 95% CI: 0.97-7.18; *P* = .058), although the result was not statistically significant. Elevated CRP (>0.5 mg/dL) was inversely related to SP, but the association was statistically significant (adjusted OR: 0.34; 95% CI: 0.08-1.45; *P* = .145).

## Discussion

Urolithiasis is frequently encountered in clinical practice, with ureteral calculi representing a substantial proportion of urinary tract stones.^5,9,10^ Several treatment strategies are available, ranging from observation and/or MET to active treatment with ureteroscopic lithotripsy or SWL.^10,11^ Surgical applications may result in complications such as morbid ureteral injuries, despite low rates.^12^ Therefore, observing for SP in selected patients is an important part of the approach algorithm for ureteral stones. Consequently, identifying suitable candidates for SP is an important part of treatment planning.

In the present prospective study, smaller stone sizes and areas, lower HU values, soft stone consistency, hematuria, and lower rates of elevated CRP were related to SP of ureteral stones in univariable analysis. In contrast, stone location, stone side, degree of hydronephrosis, serum leukocyte count, and stone composition were not significantly associated with SP. Among these findings, the evaluation of stone composition is particularly noteworthy, as few studies have directly investigated its relationship with spontaneous ureteral stone passage. Our findings suggest that physical characteristics of the stone, particularly its size and density, may be more relevant to SP than its chemical composition.

SP remains an important consideration in the management of appropriately selected patients with ureteral stones, as active interventions, such as ureteroscopy, although highly effective, are not without potential complications. Stone size is one of the most consistently reported determinants of SP, with the probability of passage decreasing as stone size increases.^3,4,11-18^ Consistent with previous studies, both stone size and stone area were significantly lower in patients who experienced SP in our cohort. The larger contact surface of a stone may increase resistance to its progression through the ureter, providing a plausible explanation for the observed association between greater stone burden and failure of SP.

Relatively few studies have examined factors associated with spontaneous stone passage. Sfoungaristos et al, in their study of 156 cases, showed that an increased WBC count can be an important marker that increases the possibility of SP.^5^ In another study involving 114 patients, Sfoungaristos et al revealed similar findings and showed that serum WBC count may be an important parameter.^14^ However, in the study conducted by Shah et al, no correlation was found between the serum leukocyte count at initial presentation and SP.^15^ Similarly, serum leukocyte count was not associated with stone passage in our cohort.

Another notable marker for predicting stone passage is CRP. The CRP level, which is a systemic inflammatory marker, may increase due to the local irritative effects of the stone in the ureter.^16,17^ Park et al reported the CRP level to be significantly lower in patients with ureteral stones smaller than 8 mm and who had passed their stones within 8 weeks.^16^ In their study of 185 patients, Jain et al identified elevated CRP levels as a negative predictive factor of passage in patients with 5-10 mm distal ureteral stones.^17^ Conversely, a multicenter retrospective cohort study found no association between CRP at initial presentation and spontaneous stone passage in multivariate analysis.^15^ In our study, an inverse relationship was found between a high CRP level (>0.5 mg/dL) and SP of ureteral stones. In multivariate analysis, an elevated CRP level showed an inverse but non-significant association with SP.

Previous studies have identified stone location as a factor associated with SP, with higher passage rates generally reported for distal than proximal ureteral stones.^15,18,19^ In our study, distal ureteral stones were also more frequent in the SP group than in the NSP group; however, this difference did not reach statistical significance. Thus, although the distribution observed in our cohort was directionally consistent with previous reports, no significant relationship between stone location and SP was demonstrated.

There are a limited number of studies evaluating stone side and showing significant differences. In a study conducted by Miller and Kane in 1999, it was reported that the probability of SP is higher in right ureteral stones.^20^ On the other hand, Sfoungaristos et al reported that left-sided stones were more likely to pass spontaneously, and this difference was based on the anatomical features of the ureters and their neighborhood.^14^ Contrary to both studies, our study showed that stone side had no effect on the passage, in agreement with other studies in the literature.^15^

One of the most clinically important findings of obstruction is hydronephrosis. As reported in the literature, stone size directly affects the development and degree of hydronephrosis.^21^ Studies have shown that the presence of hydronephrosis is a negative predictive factor for SP compared with its absence.^13,15,22^ When our own data were evaluated, it was detected that hydronephrosis had no effect on SP in ureteral stones. One possible explanation is that the stone may not have caused complete urinary obstruction at the time of diagnosis. Since we do not have information about the pressure values in the proximal part of the stone, it is not possible to reach a clear judgment on this topic.

According to our data, the HU value of the stone was found to be lower in the group that experienced spontaneous stone passage. Selvi et al revealed that there was no association between stone HU values and spontaneous stone passage.^22^ In a recent study, MET was given to all patients and lower HU values were reported in those with SP.^23^ However, Erturhan et al showed that HU and stone density did not affect the success of MET.^24^ In light of our data, it was also seen that the mechanical resistance of stones falling with SP was lower and they were broken more easily.

Most of the ureteral stones in the population are calcium oxalate or mixed-type stones containing calcium oxalate. Naturally, this numerical difference is statistically reflected in the spontaneous stone passage. Although the number of calcium-based stones was slightly higher in our study, there was no statistical difference between stone types in terms of spontaneous stone passage. In a regional study conducted in Spain, it was determined that the rate of stones containing CaOx (calcium oxalate) was higher in patients that experienced SP. However, in the same study, there was no control group that could not pass stones.^25^ In a retrospective 33-year analysis by Keller et al, high rates of CaOx stones were reported in patients with spontaneous stone passage. This study also did not have a control group.^26^ In another study, Ohkawa et al found that the number of CaOx stones was high in the spontaneous stone passage group.^27^ It was shown that the calcium oxalate monohydrate stone size was smaller, whereas the size of struvite stones was larger and the probability of passage of struvite stones is lower.^27^ Unlike our study, an upper limit for stone size was not determined, and kidney stones were not excluded.

Stone composition has rarely been investigated as a determinant of spontaneous ureteral stone passage. To the best of our knowledge, no previous study has evaluated the effect of stone composition on SP by directly comparing patients with and without SP. Although calcium oxalate stones were the most common stone type in both groups, stone composition itself was not associated with SP. This finding suggests that physical stone characteristics, such as size, density, and consistency, may play a more important role than chemical composition alone.

We acknowledge that MET seems to be efficacious for ureteral stones amenable to conservative management, especially for stones >5 mm. However, administering a MET was not preferred to be used in the study group, which can be considered a limitation. However, the effect of the MET is not equal in different parts of the ureter and also there was a risk of hypotension or syncope in some patients. So, we did not administer MET in this prospective study.

Several limitations of the present study should be acknowledged. First, our study included a relatively modest number of patients from a single center. Second, stone composition could only be evaluated in patients from whom a stone specimen was successfully retrieved. Consequently, exclusion of patients with unavailable stone analysis or missing data after follow-up may have introduced selection bias. Third, although low-dose non-contrast CT was used in the vast majority of patients, a small proportion underwent evaluation with KUB and US, resulting in some heterogeneity in imaging assessment. Finally, potential variability in analgesic requirements and patient adherence to hydration recommendations could not be fully standardized. Nevertheless, the prospective design and the simultaneous evaluation of multiple clinical, laboratory-, imaging-, and stone-related characteristics, including stone composition, represent important strengths of the study.

In conclusion, a smaller stone size was found to be independently associated with the SP of ureteral stones. In univariate analyses, stone area, HU value, stone consistency, hematuria, and CRP level were found to be associated with SP; however, no independent associations were identified after adjustment. Stone composition, however, was not found to be associated with SP. These findings emphasize that stone size is an important parameter in patient selection for conservative treatment and suggest that physical stone characteristics may be more strongly associated with SP than chemical composition.

## References

[1] Pearle MS Antonelli JA Lotan Y. Urinary lithiasis: etiology, epidemiology, and pathogenesis. In: Partin AW Kavoussi LR Peters CA, eds. Campbell-Walsh-Wein Urology. 12th ed. Elsevier; 2021:2005-2035.

[2] Solakhan M Seckiner SU Seckiner I. A neural network-based algorithm for predicting the spontaneous passage of ureteral stones. Urolithiasis. 2020;48(6):527-532. (doi: 10.1007/s00240-019-01167-5) 31667542

[3] Demehri S Steigner ML Sodickson AD Houseman EA Rybicki FJ Silverman SG. CT-based determination of maximum ureteral stone area: a predictor of spontaneous passage. AJR Am J Roentgenol. 2012;198(3):603-608. (doi: 10.2214/AJR.11.7276) 22357999

[4] Skolarikos A Jung H Neisius A et al. EAU Guidelines on Urolithiasis. EAU Guidelines Office; 2026.

[5] Sfoungaristos S Kavouras A Katafigiotis I Perimenis P. Role of white blood cell and neutrophil counts in predicting spontaneous stone passage in patients with renal colic. BJU Int. 2012;110(8 pt B):E339-E345. (doi: 10.1111/j.1464-410X.2012.11014.x) 22372435

[6] Aldaqadossi HA. Stone expulsion rate of small distal ureteric calculi could be predicted with plasma C-reactive protein. Urolithiasis. 2013;41(3):235-239. (doi: 10.1007/s00240-013-0551-1) 23468212

[7] Cilesiz NC Ozkan A Kalkanli A et al. Can serum procalcitonin levels be useful in predicting spontaneous ureteral stone passage? BMC Urol. 2020;20(1):42. (doi: 10.1186/s12894-020-00608-3) PMC7168945 32306948

[8] Lee KS Ha JS Koo KC. Significance of neutrophil-to-lymphocyte ratio as a novel indicator of spontaneous ureter stone passage. Yonsei Med J. 2017;58(5):988-993. (doi: 10.3349/ymj.2017.58.5.988) PMC5552654 28792143

[9] Prakash P Agarwal S Sarpal R Dogra AK Prakash A. Composition and biochemical determinants of renal stones: a comprehensive analysis of urinary and serum mineral profiles. Urol Res Pract. 2026;52:1-6. (doi: 10.5152/tud.2026.26015) PMC13242086 42364224

[10] Abdelgawad AH Elhawy MA Hussein AAHF Fathelbab TK Tolba AKR Fawzy AM. Retrograde intrarenal renal surgery versus supine mini percutaneous nephrolithotripsy in treatment of renal pelvic stones less than 2 cm, randomized clinical study. Urol Res Pract. 2026;51(6):230-236. (doi: 10.5152/tud.2026.25121) PMC13034001 41942405

[11] Ali A Tuncel A. Üreter taşları için medikal ekspulsif tedavi. Turk J Urol. 2010;36(3):302-308. (doi: 10.5152/tud.2010.034)

[12] Öğreden E Oğuz U Demirelli E et al. Categorization of ureteroscopy complications and investigation of associated factors by using the modified Clavien classification system. Turk J Med Sci. 2016;46(3):686-694. (doi: 10.3906/sag-1503-9) 27513242

[13] Özcan C Aydoğdu O Senocak C et al. Predictive factors for spontaneous stone passage and the potential role of serum C-reactive protein in patients with 4 to 10 mm distal ureteral stones: a prospective clinical study. J Urol. 2015;194(4):1009-1013. (doi: 10.1016/j.juro.2015.04.104) 25963189

[14] Sfoungaristos S Kavouras A Perimenis P. Predictors for spontaneous stone passage in patients with renal colic secondary to ureteral calculi. Int Urol Nephrol. 2012;44(1):71-79. (doi: 10.1007/s11255-011-9971-4) 21544652

[15] Shah TT Gao C Peters M et al; British Urology Researchers in Surgical Training (BURST) Collaborative MIMIC Study Group. Factors associated with spontaneous stone passage in a contemporary cohort of patients presenting with acute ureteric colic: results from the Multi‐centre cohort study evaluating the role of Inflammatory Markers In patients presenting with acute ureteric Colic (MIMIC) study. BJU Int. 2019;124(3):504-513. (doi: 10.1111/bju.14777) 31001912

[16] Park CH Ha JY Park CH Kim CI Kim KS Kim BH. Relationship between spontaneous passage rates of ureteral stones less than 8 mm and serum C-reactive protein levels and neutrophil percentages. Korean J Urol. 2013;54(9):615-618. (doi: 10.4111/kju.2013.54.9.615) PMC3773592 24044096

[17] Jain A Sreenivasan SK Manikandan R Dorairajan LN Sistla S Adithan S. Association of spontaneous expulsion with C-reactive protein and other clinico-demographic factors in patients with lower ureteric stone. Urolithiasis. 2019;48(2):1-6. (doi: 10.1007/s00240-019-01137-x) 31025078

[18] Tchey DU Ha YS Kim WT Yun SJ Lee SC Kim WJ. Expectant management of ureter stones: outcome and clinical factors of spontaneous passage in a single institution’s experience. Korean J Urol. 2011;52(12):847-851. (doi: 10.4111/kju.2011.52.12.847) PMC3246518 22216398

[19] Sáenz Medina J Alarcón Parra RO Redondo González E et al. Prognostic factors of spontaneous expulsion in ureteral lithiasis. Actas Urol Esp. 2010;34(10):882-887. (doi: 10.1016/s2173-5786(10)70218-3) 21159285

[20] Miller OF Kane CJ. Time to stone passage for observed ureteral calculi: a guide for patient education. J Urol. 1999;162(3 pt 1):688-691; discussion 690-691. (doi: 10.1097/00005392-199909010-00014) 10458343

[21] Goertz JK Lotterman S. Can the degree of hydronephrosis on ultrasound predict kidney stone size? Am J Emerg Med. 2010;28(7):813-816. (doi: 10.1016/j.ajem.2009.06.028) 20837260

[22] Selvi I Baydilli N Tokmak TT Akinsal EC Basar H. CT-related parameters and Framingham score as predictors of spontaneous passage of ureteral stones ≤ 10 mm: results from a prospective, observational, multicenter study. Urolithiasis. 2021;49(3):227-237. (doi: 10.1007/s00240-020-01214-6) 32980972

[23] Kachroo N Jain R Maskal S et al. Can CT-based stone impaction markers augment the predictive ability of spontaneous stone passage? J Endourol. 2021;35(4):429-435. (doi: 10.1089/end.2020.0645) 32883100

[24] Erturhan S Bayrak O Mete A Seckiner I Urgun G Sarica K. Can the Hounsfield unit predict the success of medically expulsive therapy? Can Urol Assoc J. 2013;7(11-12):e677-e680. (doi: 10.5489/cuaj.352) PMC3840511 24282456

[25] Muñoz-Velez D Garcia-Montes F Costa-Bauza A Grases F. Analysis of spontaneously passed urinary tract stones. Urol Res. 2010;38(1):35-39. (doi: 10.1007/s00240-009-0230-4) 19949784

[26] Keller EX De Coninck V Audouin M Doizi S Daudon M Traxer O. Stone composition independently predicts stone size in 18,029 spontaneously passed stones. World J Urol. 2019;37(11):2493-2499. (doi: 10.1007/s00345-018-02627-0) 30719571

[27] Ohkawa M Tokunaga S Nakashima T Yamaguchi K Orito M Hisazumi H. Spontaneous passage of upper urinary tract calculi in relation to composition. Urol Int. 1993;50(3):153-158. (doi: 10.1159/000282474) 8465482

